# High grade angiosarcoma arising in fibroadenoma

**DOI:** 10.1186/1746-1596-6-125

**Published:** 2011-12-20

**Authors:** Emina Babarović, Gordana Zamolo, Elvira Mustać, Miroslav Strčić

**Affiliations:** 1 Department of Pathology, School of Medicine, University of Rijeka, Braće Branchetta 20, Rijeka, Croatia; 2 Department of General Surgery, Rijeka University Hospital Center, Krešimirova 42, Rijeka, Croatia

**Keywords:** Breast, fibroadenoma, primary angiosarcoma, mastectomy

## Abstract

Primary angiosarcoma of the breast is a rare tumour that account for fewer than 0.05% of all malignant mammary tumours. Angiosarcoma may have an perfidious clinical onset. Radiologic findings are often nonspecific and may appear completely normal in one-third of cases with primary angiosarcoma. The prognosis is usually poor because of the high rates of local recurrence and early development of metastases. Aggressive surgical resection is the mainstay of treatment. The role of adjuvant therapy has not yet been well established.

Here we present a case of a 53 year old, postmenopausal women with primary angiosarcoma arising in fibroadenoma. To our knowledge, this is the first case described in the literature to date.

## Background

Angiosarcoma is a rare malignancy of endovascular origin. Skin and superficial soft tissue are the most common locations that become involved. This is in contrast with the deeper location of other soft tissue sarcomas. Less frequently it can occur in various organs and has been reported in uterus [1], ovary [2], small intestine [3], lung [4], heart [5], oral cavity [6], orbit [7] and thyroid [8]. Unlike angiosarcomas of the skin, deep angiosarcomas more commonly have an epitheloid appearance consisting of nests and clusters of round cells of high nuclear grade. These so called epitheloid angiosarcomas consists of sheets of highly atypical round cells with prominent nuclei, some of which contain intracytoplasmic lumens.

Primary soft tissue sarcomas represent fewer than 1% of primary breast malignancies. Angiosarcomas are rare and highly aggressive tumours that account for fewer than 0.05% of all malignant mammary tumours [9]. They may arise spontaneously (as a primary malignancy) or as a sequel of radiation therapy and postoperative lymphedema due to breast cancer (secondary). In comparison, primary angiosarcomas are relatively rarer, they tipically occurs in younger women and arise from breast parenchyma. Secondary angiosarcomas usually occurs in elderly women, arise from skin and show a pattern of infiltration into breast parenchyma from skin and subcutis. Both primary and secondary breast angiosarcoma carry a prognosis worse than mammary carcinoma [10]. Synchronous bilateral angiosarcoma has been reported [11], although the contralateral breast is a common site of metastasis and such cases may potentially represent metastatic spread. Primary breast angiosarcoma has also been described in conjunction with synchronous primary breast carcinoma [12,13] and also with silicone granuloma after breast augmentation [14].

Fibroadenomas are the most common benign tumors of the female breast. They are most frequent in young women, mainly those under 30 years, but may be seen at any age. These tumours are characterized by a proliferation of both stromal and epithelial elements of the breast. Fibroadenoma may be associated with fibrocystic changes, calcifications, proliferative epithelial changes, and extremely rarely, lobular and ductal non-invasive and invasive carcinoma may occur within fibroadenoma [15].

Angiosarcoma arising in fibroadenoma has not been documented so far, to the best of our knowledge. Only one case of angiosarcoma arising in a recurrent phyllodes tumor has been published [16]. Here we present a case of a 53 year old women with primary angiosarcoma arising in fibroadenoma.

## Case presentation

A 53-year old postmenopausal female presented with a lump in the upper outer quadrant of her right breast. At physical examination a mass was palpable, painless, firm and not fixed to the underlying structure. There was no history of pain or nipple discharge. Overlying skin was unremarkable. The patient had not received any radiotherapy in the past. Any family history of breast malignancies was denied.

Mammography revealed two well circumscribed nodular formations in the upper outer quadrant of right breast. The contours were smooth, with high density and homogeneous. The conclusion of radiologist was benign finding (BI-RADS 2).

**Figure 1:**
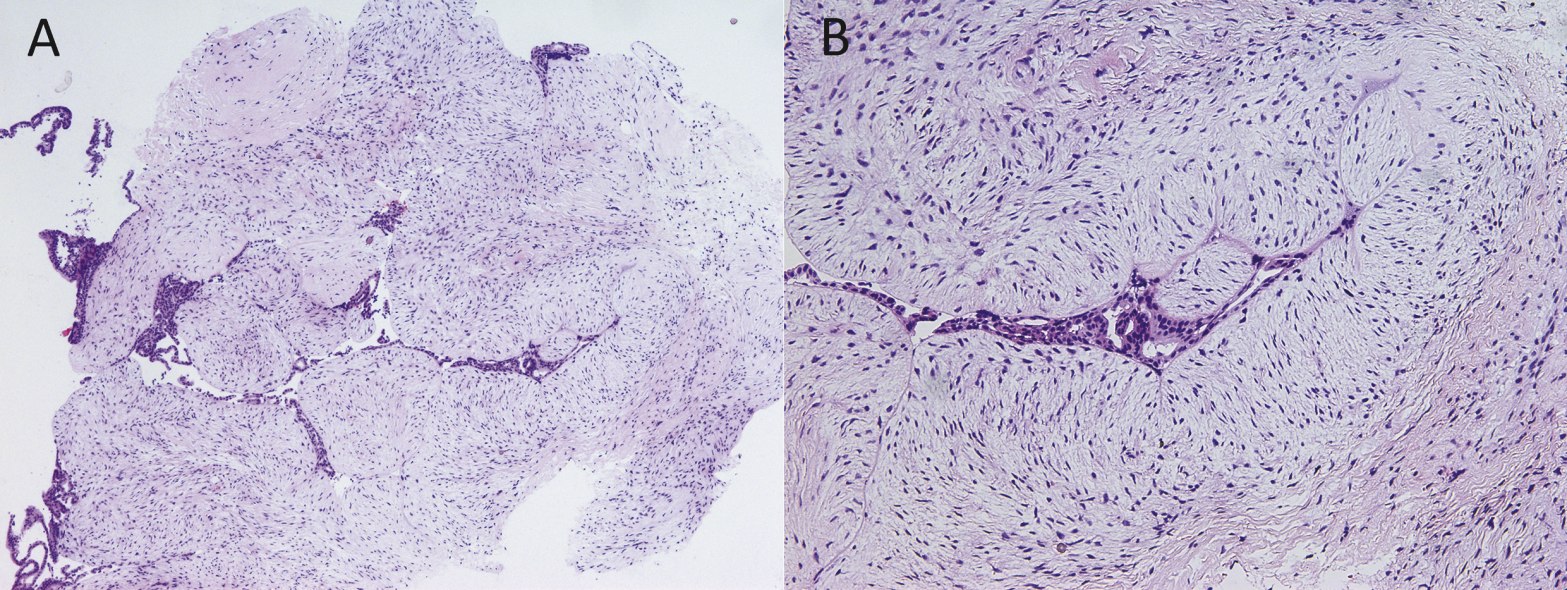
**Core needle biopsy specimen of the breast showing fibroadenoma**. (HE), 40 × (A), 100 × (B).

Ultrasonography revealed macrolobulated complex formation measuring in diameter 41 mm. The ultrasonography guided core needle biopsy was also taken. Pathologic examination of this sample showed fibroadenoma (Figure 1).

After 6 months, a lump in breast progressively increased in size to a size of 80 × 35 mm. The patient did not complain of tenderness or pain. There was no history of nipple retraction or discharge. Overlying skin was unchanged. No axillary or supraclavicular lymph nodes were palpable. The contralateral breast and axilla were normal.

**Figure 2:**
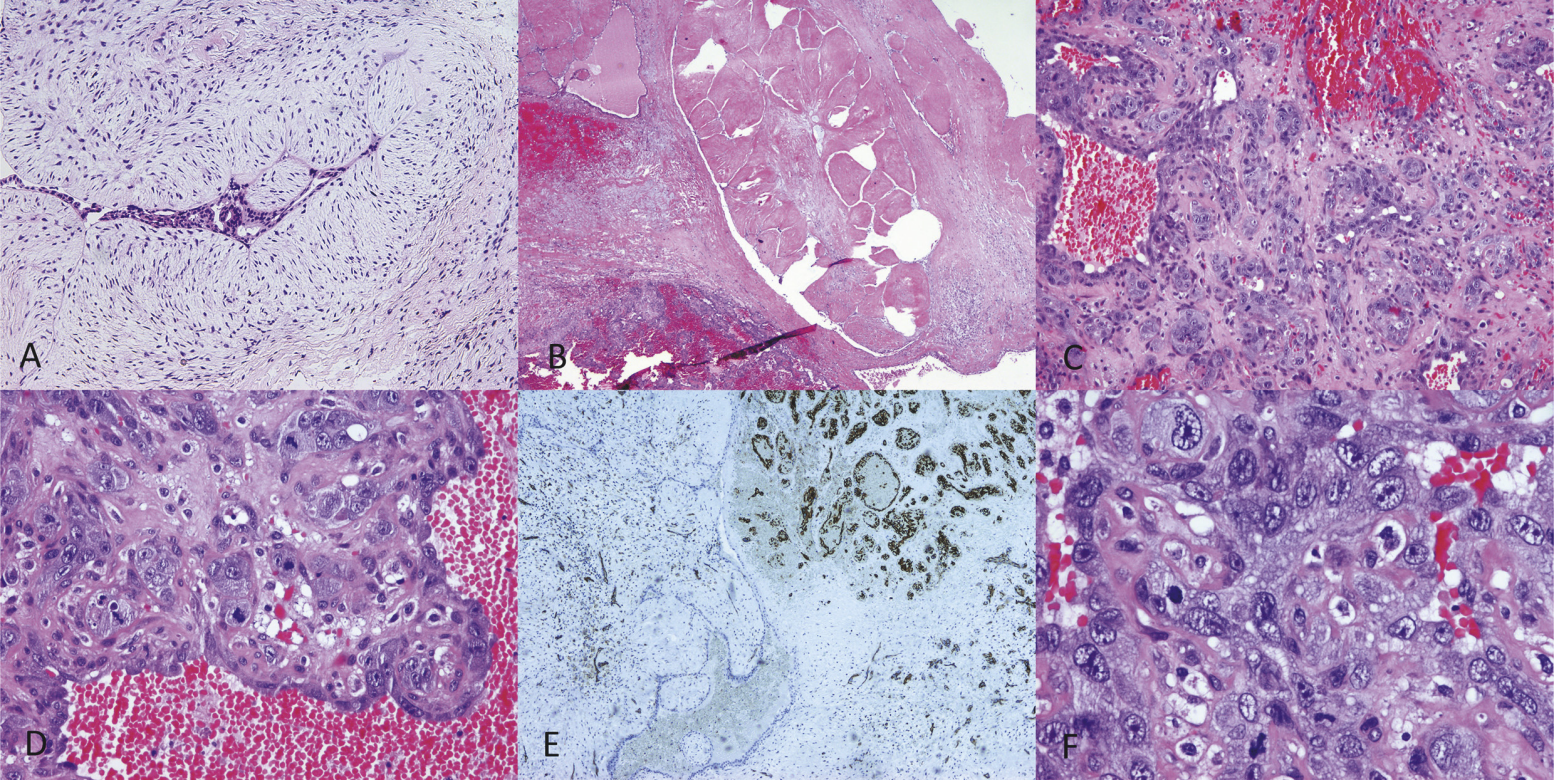
**Microscopic features of the resected tumor**. Fibroadenoma of the breast HE, 100 × (A). Fibroadenoma and angiosarcoma in the same field, HE, 20 × (B). Angiosarcoma, the tumor is highly vascular with relatively solid spindle cell proliferation and area of stromal hemorrhage, HE, 100 × (C). Prominent tufts and papilations composed of enlarged highly malignant endothelial cells with brisk mitotic activity, HE, 200 × (D). Immunohistochemical stain for vascular marker CD34 was positive in angiosarcoma and negative in fibroadenoma, 40 × (E). Highly malignant endothelial cells, HE, 400 × (F).

**Figure 3:**
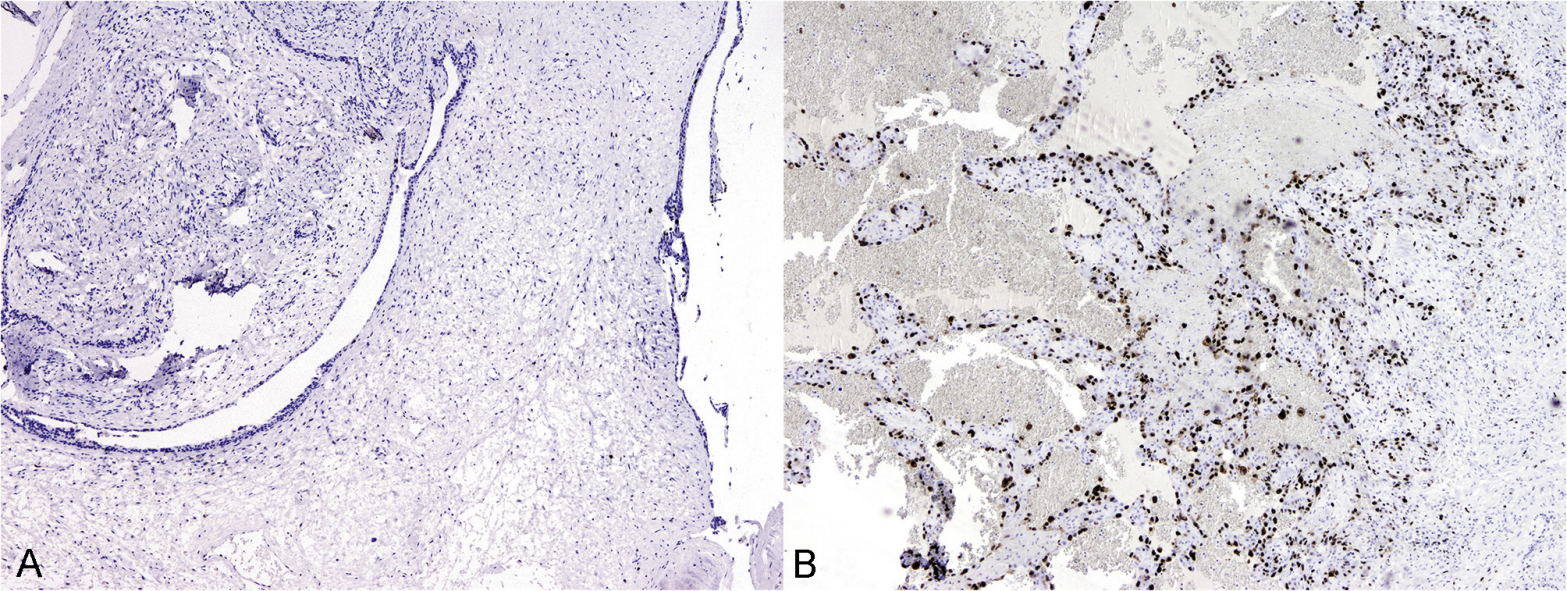
**Immunohistochemical staining with monoclonal antibody Ki-67/MIB-1**. Ki-67 proliferative activity in fibroadenoma, 40 × (A); and in angiosarcoma, 40 × (B).

Lumpectomy was suggested. On gross examination, the specimen consisted of a fibrofatty piece of tissue measuring 5 × 5 × 4.5 cm. On sectioning there was a lobulated, partly soft and hemorrhagic, incapsulated nodule measuring 4 cm in diameter. Microscopy revealed a tumor to be a fibroadenoma with pericanalicular and intracanlicular patterns of growth and with stromal hyalinization. Within the tumor there was one focus composed of marked proliferation of poorly formed, thin-walled vascular channels lined with highly atypical endothelial cells. Necrotic foci with marked hemorrhage and relatively solid proliferation of polygonally-shaped and spindle atypical endothelial cells were seen. Prominent tufts and papilations composed of enlarged, polygonally-shaped, highly malignant endothelial cells, with brisk mitotic activity, was also observed (Figure 2). The endothelial cells had abundant cytoplasm with spindle-shaped or round nuclei and showed either a papillary or solid growth pattern. A panel of immunohistochemical markers was performed. The neoplastic endothelium was diffusely and strongly reactive for CD31. With CD34 immunostain parts of the tumor were strongly positive (Figure 2), while in poorer differentiated areas, with epitheloid and solid appearance almost completely negative. The neoplastic cells were negative for pan cytokeratin clone MNF 116 and desmin. In addition, proliferative activity was determined using monoclonal antibody Ki-67/MIB-1. Ki-67 proliferative activity in fibroadenoma was 4% and in part of the lesion with malignant endothelial cells 57% (Figure 3). A diagnosis of high-grade angiosarcoma arising in fibroadenoma was made. Radical mastectomy was performed subsequently and no residual tumour was found. Cysts with epithelial lining showing apocrine metaplasia, sclerosing adenosis with microcalcifications and additional fibroadenoma measuring 3 mm in diameter was observed in the adjacent breast tissue.

The postoperative course was uneventful. After the operation extensive clinical evaluation was performed to exclude potential metastatic processes. All test results were normal. Eight months after the surgery the patient is free of recurrence and metastasis.

## Discussion

Angiosarcoma of the breast, including primary and secondary type, remain rare malignancy. Although, primary angiosarcomas may arise at any location in the body, they rarely arise from major vessels and have a predilection for skin and supreficial soft tissue. When it occurs in the breast, it affects women during the third and fourth decades of life. The etiology of primary angiosarcoma is still unresolved. Twelve percent of the cases are found during pregnancy, implaying a hormonal effect [17]. In contrary our patient was slightly older post-menopausal women. There are no known risk factors for developing primary angiosarcomas, but it has been reported adjacent to foreign materials. There has been a report of development breast angiosarcoma adjacent to silicone granuloma in a patient who underwent breast augmentation with silicone implants [14]. An extuberant host response in the form of a fibrous tissue capsule around the foreign material may represent an important intermediate step in the development of the angiosarcoma. The prognosis of primary angiosarcoma is usually poor because of the high rates of local recurrence and early development of metastases [18]. Angiosarcoma of the breast has a tendency to metastasize hematogenously, the regional lymph node involvement is uncommon either at presentation or at recurrence [19]. The most common sites of metastasis are lung, bone, liver and skin. Angiosarcoma may have an perfidious clinical onset, presenting as a painless often discrete mass that grows rapidly. Radiologic findings are often nonspecific, and angiosarcoma may easily be overlooked. Mammograms may show a nonspecific mass and may appear completely normal in one-third of cases with primary angiosarcoma. Visible masses may be ill-defined or circumscribed, and may be round to oval or more lobulated. Associated coarse calcifications may be present [20]. Luini et al reported that most authors report normal mammographic findings in cases of primary angiosarcoma [9]. Ultrasonography tends to be nonspecific. The diagnosis of primary angiosarcoma of the breast may be challenging. The main differential diagnoses of high-grade angiosarcoma are metaplastic carcinoma with angiosarcomatous differentiation and other poorly differentiated sarcomas. Histologically, the presence of characteristic lower-grade angiosarcoma at the periphery of the lesion can be helpful in diagnosis. In our case peripheral parts of tumor were composed of poorly formed, thin-walled vascular channels lined with highly atypical endothelial cells. Similarly, metaplastic carcinomas may be associated with DCIS or areas of typical invasive carcinoma of ductal type. The use of immunohistochemical markers for endothelium, CD31, CD34 and factor VIII, confirm most angiosarcomas including poorly differentiated ones. As in our case, usually in angiosarcomas CD34 highlights mainly the uninvolved background vessels, and in fact, there are different reports that have shown variable reaction patterns to CD34 ranging from completely negative to approximately 80% positivity of the neoplastic cells [21]. In our patient, the intense reaction was seen in peripheral areas of neoplastic lasion and in uninvolved vasculature, while in poorer differentiated, solid areas there was no immunoreactivity with CD34. CD31 is the most specific marker for endothelial differentiation, whereas CD34 is more sensitive. In our case a diagnosis of high grade angiosarcoma was confirmed by strong and diffuse positive reaction for CD31, along with negative cytokeratin and desmin stains. So the diagnosis of metaplastic carcinoma and possible combined myovascular lesions were ruled out. Moreover, a Ki-67 proliferative score of 57% along with marked pleomorphisam of endothelial cells are striking features in favour of high grade malignancy.

Primary breast angiosarcomas are a heterogeneous group of lesions histologically, composed of interanastomosing vascular channels lined by hyperchromatic endothelial cells, occurring within the breast parenchyma. Angiosarcomas of the breast can be classified into three main growth patterns, well-differentiared (Grade I) to poorly differentiated (Grade III) tumors. The cells of the low grade angisarcoma resemble endothelium, while in majority of high grade angisarcoma endothelial tufting and papillary formations are prominent and the endothelial cells show marked cytological atypia with prominent nucleoli and frequent mitotic figures as in our case. Haemorrhage into the surrounding stroma („blood lakes") and areas of necrosis may be present. However, variations in growth pattern are commonly found within a single lesion; in particular, high-grade lesions can have lower-grade areas at their peripheral and superficial aspects. It was believed that histologic grade is of prognostic importance, but a recent study show that there is no correlation between histologic grade and patients outcome [22]. The prognostic factors of breast angiosarcoma include the tumor size, presence of residual disease, cellular pleomorphism and proliferative index [23,24]. Aggressive surgical resection is the mainstay of treatment. Even tought angiosarcoma at other sites often involve regional lymph nodes [25], in breast angiosarcoma the regional lymph node involvement is uncommon either at presentation or at recurrence [19,25,26]. The role of adjuvant therapy with radiotherapy and chemotherapy has not yet been well established. A recent study reported a case of complete response to adjuvant thalidomide therapy [27]. The aggressive nature of this disease require exploration of new adjuvant therapy regimens.

## Conclusions

The rarity of primary angiosarcoma, unusual presentation of our case and possible diagnostic pitfalls associated with poor prognosis emphasizes the need for systemic presentation of these tumors in order to help pathologists and clinicians to know that such an entity can initially present as fibroadenoma. The aggressive nature of this disease demands careful follow-up of these patients, due to rarity of the condition the appropriate strategies of treatment remain to be determined. The question remains should we consider less aggresive terapeutic approach for small lesions diagnosed microscopically?

## Consent

Written informed consent was obtained from the patient for publication of this case report and any accompanying images. A copy of the written consent is available for review by the Editor-in-Chief of this journal.

## Competing interests

The authors declare that they have no competing interests.

## Authors' contributions

EB participated in the design of the study, and drafted the final version of the manuscript. GZ participated in design of the study and manuscript drafting, carried out histopathological evaluation. EM participated in histopathological evaluation and helped in drafting the manuscript. MS tracked the clinical data and helped in drafting the manuscript.

All authors read and approved the final manuscript.
